# Acoustic and Temporal Partitioning of Cicada Assemblages in City and Mountain Environments

**DOI:** 10.1371/journal.pone.0116794

**Published:** 2015-01-15

**Authors:** Bao-Sen Shieh, Shih-Hsiung Liang, Yuh-Wen Chiu

**Affiliations:** 1 Department of Biomedical Science and Environmental Biology, Kaohsiung Medical University, Kaohsiung, Taiwan; 2 Department of Biotechnology, National Kaohsiung Normal University, Kaohsiung, Taiwan; 3 National Museum of Marine Biology and Aquarium, Pingtung, Taiwan; University of Maribor, SLOVENIA

## Abstract

Comparing adaptations to noisy city environments with those to natural mountain environments on the community level can provide significant insights that allow an understanding of the impact of anthropogenic noise on invertebrates that employ loud calling songs for mate attraction, especially when each species has its distinct song, as in the case of cicadas. In this study, we investigated the partitioning strategy of cicada assemblages in city and mountain environments by comparing the acoustic features and calling activity patterns of each species, recorded using automated digital recording systems. Our comparison of activity patterns of seasonal and diel calling revealed that there was no significant temporal partitioning of cicada assemblages in either environment. In addition, there was no correlation between the acoustic distance based on spectral features and temporal segregation. Heterospecific spectral overlap was low in both city and mountain environments, although city and mountain cicada assemblages were subject to significantly different levels of anthropogenic or interspecific noise. Furthermore, for the common species found in both environments, the calling activity patterns at both seasonal and diel time scales were significantly consistent across sites and across environments. We suggest that the temporal calling activity is constrained by endogenous factors for each species and is less flexible in response to external factors, such as anthropogenic noise. As a result, cicada assemblages in city environments with low species diversity do not demonstrate a more significant temporal partitioning than those in mountain environments with high species diversity.

## Introduction

Resource partitioning has long been a central concept in studying ecological communities [1]. For animals that call loudly to attract mates, their sexual sounds must traverse a common space, where they suffer acoustic interference from other sympatric species. Therefore, the partitioning of acoustic resources in closely related sympatric species is crucial for reproductive function. Various strategies are used to avoid the negative effects of acoustic competition, including the partitioning of acoustic features or partitioning of calling in time and space [2].

Evidence for acoustic partitioning has been found in many animal assemblages, such as those of frogs [3, 4], birds [5, 6], and crickets [7, 8]. For animals that have their peak breeding season at the same time of year and show no evidence for the partitioning of calling time on a diel scale, interspecific masking might effectively be reduced by the partitioning of acoustic features [6]. Nevertheless, Chek et al. [3] suggested that partitioning in time and in space were the most important strategies to reduce interspecific acoustic competition.

Temporal activity patterns indicate how species utilize the environment along a time scale, and they are considered to represent an important niche dimension [9]. As a resource axis, time can be partitioned on both the diel and seasonal scales [10, 11]. The partitioning of calling activity in time has been found in many frog assemblages and cicada assemblages [12, 13, 14].

Cicadas use loud calling songs to attract mates over long distances [15]. They are excellent species for investigating acoustic and temporal partitioning: male cicadas call with loud and conspicuous songs only for mate attraction, each species has its own specific calling songs [16–18], and cicadas provide good study examples of multi-species acoustic communities in insects [14, 18]. Temporal partitioning in cicadas may facilitate coexistence through avoidance of direct acoustic interference. Different animal species tend to have taxon-specific diel activity patterns and are evolutionarily constrained in their diel activity patterns [19]. In addition to diel calling activity, temporal partitioning may also be effective on a seasonal scale [20]. Which scale is more evident in temporal partitioning of cicada assemblages, seasonal or diel? Do cicada species exhibit environment variation in seasonal or diel activity patterns? Is temporal activity a species characteristic, or do species change the timing of their activity based on the species composition of local cicada assemblages? Few studies have compared the temporal partitioning of cicada assemblages in different environments to answer these questions.

Anthropogenic noise and its impact on animals have drawn increasing attention because of rapidly expanding urbanization [21–25]. Urban noise has been recognized having profound effects not only on single species [23] but also on interspecific interactions [26] and community structure [24, 27, 28]. Recent urban noise studies have primarily focused on vertebrates. In addition, the few urban noise studies on invertebrates have been conducted only on the species levels [29, 30]. Morley et al. [25] suggested that studies on invertebrates and their community structure in particular should receive greater emphasis because of their high potential to respond to urban noise. In this study, we compared the temporal patterns of cicada calling activity in two contrasting environments, a city environment with high anthropogenic noise and a mountain environment with low anthropogenic noise, by using automated digital recording systems. We investigated the partitioning of cicada assemblages in these two different environments from a hierarchical viewpoint, from seasonal partitioning and diel partitioning to spectral feature partitioning. We hypothesized that sympatric cicada species that overlap more in their calling season should partition their diel activity, whereas those that segregate their calling season should not partition their diel activity. To reduce acoustic interference from other sympatric species, higher acoustic dissimilarities or distances were expected in sympatric species with higher similarities or overlaps in seasonal and diel calling activity, and vice versa; that is, a positive relationship was expected between the distances of spectral features and similarities of seasonal and diel calling activity for pairwise sympatric species. We also hypothesized that diel activity patterns were species-specific and more constrained, whereas seasonal activity patterns were not. That is, species found in different environments would have a common diel activity pattern, while their seasonal activity patterns might change.

## Methods

### Ethic statement

This study was non-invasive research and was in compliance with the legal requirements of the country in which it was conducted. Permission to conduct research at the mountain site was received from the Shanping Forest Ecological Garden of the Taiwan Forestry Research Institute. Permissions to conduct research at the city sites were received from the Tourism Bureau of Kaohsiung City Government and Taiwan Water Corporation.

### Study site and audio recording

We used an automated digital recording system, an omni-directional microphone (Mipro 707P) connected to an amplifier (ROLLS MX54s) and a Genuine desktop computer (BK888-I1 Core2 Duo 2.93GHz) to record cicada calling songs at three study sites in two environments in Taiwan from 2009–2011: SP (Shanping site: mountain environment, N22°58′, E 120°41′, elevation 600–700 m); CC (Cheng-Ching Lake site: city environment, N22° 39′, E120° 20, elevation < 20 m); and SO (Shoushan site: city environment, N22°38′, E 120°16′, elevation < 356 m). The two city sites are approximately 8 km apart and separated by buildings, highways and roads. The mountain site is approximately 48 km away from the city sites and contains diverse flora, including natural broadleaf forests. At the beginning of the study, three sets of recording systems were placed at the SP site, and one set each at the two city sites. However, the SP site was damaged significantly by Typhoon Morakot in August of 2009, and recordings were mostly missed by two of the sets of recording systems. Therefore, we used only recordings from one set at each of the three study sites in 2011 for the temporal analysis. The recording schedule was set to make a 5-min recording every half hour from 05:30 to 19:00 every day in 2011 for the three study sites using the Total Recorder software (Professional Edition 8.2, High Criteria Inc.). A total of 28 5-min recordings were obtained per day. A 5-min recording was separated into 10 30-sec sections and checked for occurrences of calling songs of each cicada species. We divided the number of sections that contained occurrences of calling songs by 10 to represent the calling activity for each half-hour. The half-hour calling activity was then used to measure diel activity patterns and seasonal activity patterns for each cicada species. The diel activity pattern for each species (Tables A and B in S1 File, Figures A and B in S2 File) was obtained by averaging the calling activity of the same half-hour period of those days with the calling occurrences. Seasonal activity patterns were obtained by using a time series analysis of SAS Enterprise Guide 5.1 and Enterprise Miner 12.1 (SAS Institute Inc., Cary, NC, USA) software. First, daily calling activity was calculated by averaging the 28 half-hour calling activities. Seasonal (semimonthly) calling activity (Tables C and D in S1 File, Figures C and D in S2 File) was then obtained by averaging those daily calling activities of each half-month. There were no recordings due to equipment failures on the following dates: Feb. 1–12 and Aug. 12–18 for site SP, June 20–30 and July 1–7 for site SO, and Sept. 8–30 and Oct. 1–19 for site CC. We filled in the missing values using linear interpolation methods. The temporal segregation of calling activity between species was examined using pairwise Spearman rank correlations.

In addition to automated digital recording systems, a handheld device (a Denon portable DN-F20R recorder connected to a Sennheiser ME67 unidirectional microphone with sampling frequency of 48 kHz) was also used to record additional cicada calling sounds along fixed trails at the three study sites. Recording was conducted once every month during Feb.-Nov. of 2011 for the mountain SP site and during June-Nov. of 2011 for the two city sites (CC and SO). We measured the maximum noise level (dB, maximum hold function on Sound Level Meter TES-1350, TES, Taiwan, R.O.C.) and the ambient noise level (dB, Sound Level Meter TES-1350: slow response, C-weighting function) at 3 fixed locations along the fixed trails of each study site using methods described in Shieh et al. [30]. The sound level meter was held 1.5 m above the ground and on artificial trails or roads; at this height and location, anthropogenic sources such as traffic and human activity are the main sources of noise. The two city sites had significantly higher noise levels than the SP mountain site (Maximum noise level in dB: F_2,209_ = 89.3, P < 0.01; at CC site, mean ± SE = 65.72 ± 0.43, range = 59.2~76, n = 76; at SO site, 65.8 ± 0.37, 60.1~74.3, n = 76; at SP site, 57.9 ± 0.61, 48.1~69.6, n = 60) (Ambient noise level in dB: F_2,209_ = 80.2, P < 0.01; at CC site, mean ± SE = 60.8 ± 0.57, range = 30.8~73.2, n = 76; at SO site, 59.73 ± 0.63, 30.2~68.4, n = 76; at SP site, 49.85 ± 0.62, 42.5~65.6, n = 60).

### Study subjects and acoustic analysis

Cicada species were first identified by hearing according to the CD of Chen [31] and then by spectrograms. We recorded a total of 12 species at the SP mountain site from 2009–2012, but only 11 species were recorded during 2011: *Cryptotympana holsti* Distant, *Cr. takasagona* Kato, *Euterpnosia gina* Kato, *Leptosemia sakaii* (Matsumura), *Meimuna opalifera* (Walker), *Mogannia formosana* Matsumura, *Platypleura takasagona* Matsumura, *Pomponia linearis* (Walker), *Semia watanabei* (Matsumura), *Tanna taipinensis* (Matsumura), and *T. viridis* Kato. *Formotosena seebohmi* (Distant) was recorded in 2009 at the SP mountain site but not in 2011 and therefore was not included in the current analysis.Three cicada species were recorded at the two city sites: *Chremistica ochracea* (Walker), *Cr. atrata* (Fabricius), and *Cr. takasagona*. The only species found in both the mountain and city environments was *Cr. takasagona*.

Sound recordings of good quality from both the automated digital recording systems and the hand-held device were analyzed using Avisoft-SASLab Pro software (version 5.2.05) at a resolution of 16 bits. We produced spectrograms with FFT = 1024, frame size = 100%, hamming window, frequency resolution = 43 Hz, overlap = 87.5%, and time resolution = 2.9 ms.

**Figure 1:**
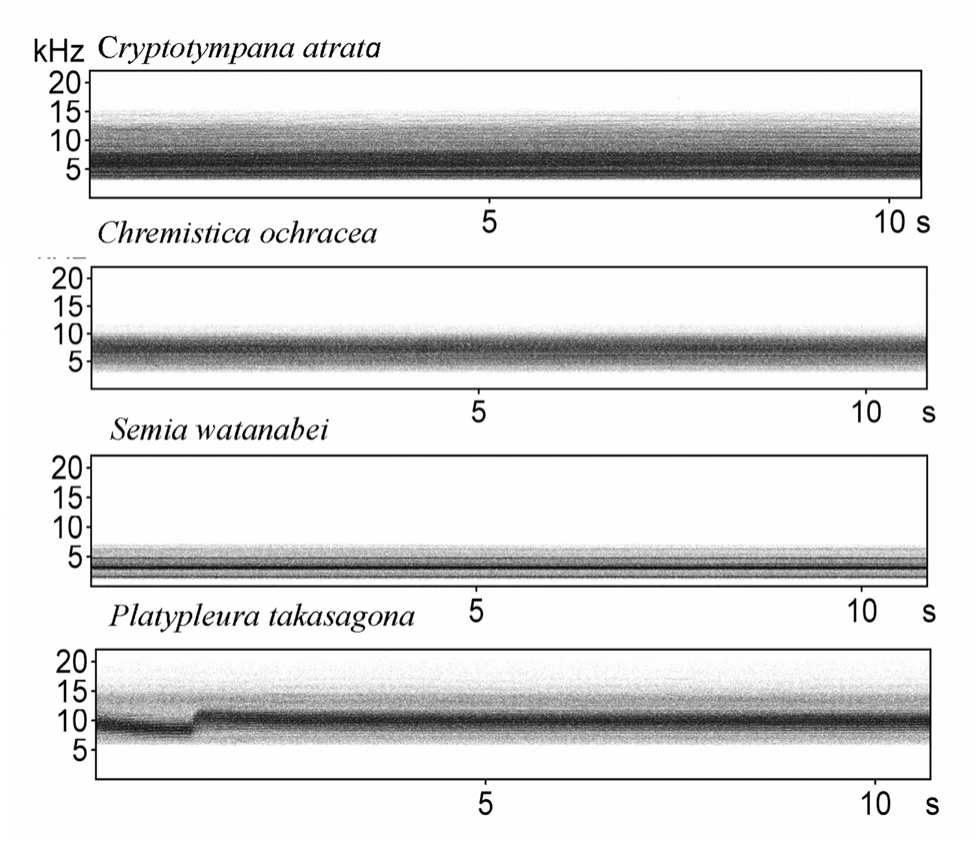
Spectrograms of cicada calling songs of tonal band patterns.

**Figure 2:**
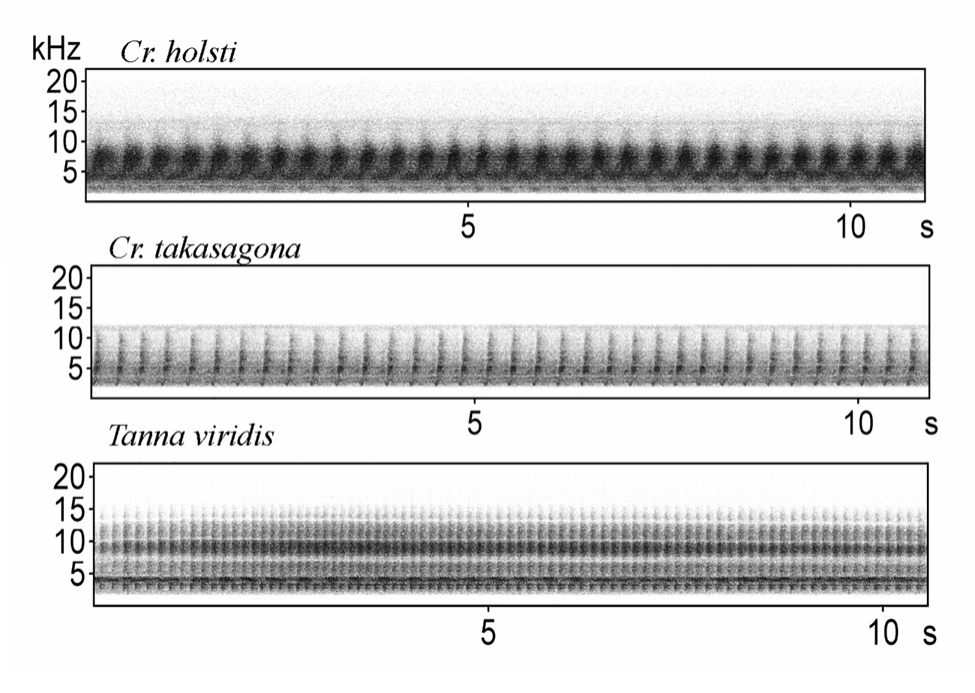
Spectrograms of cicada calling songs of pulse trains patterns.

**Figure 3:**
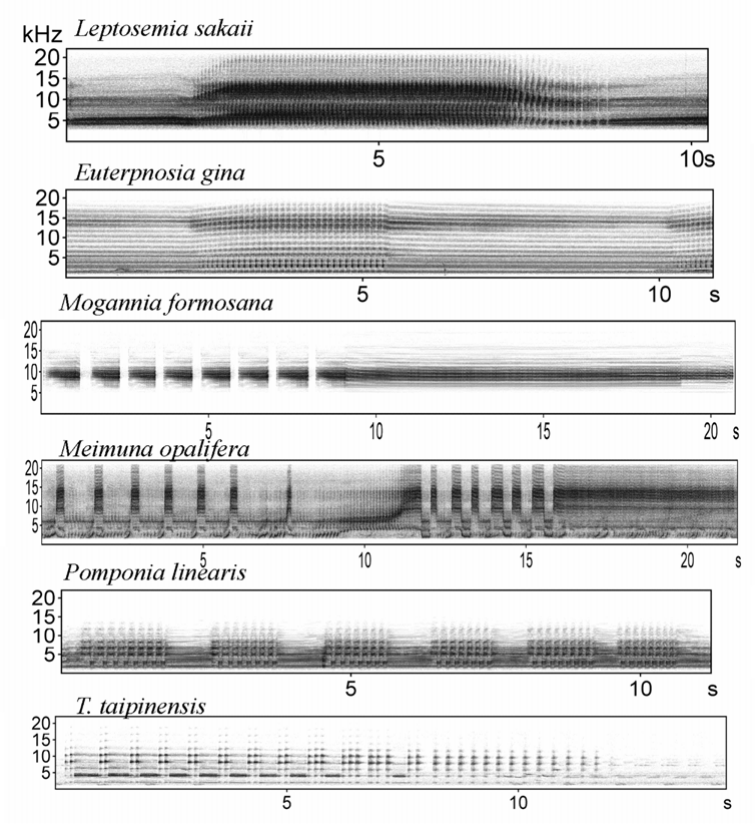
Spectrograms of cicada calling songs of mixed patterns.

All cicada calling songs were classified into three categories according to the time-frequency patterns on spectrograms: (A) tonal band patterns, which consists of continuous frequency bands only (Fig. 1); (B) pulse trains patterns, which consists of monotypic pulse notes (Fig. 2); and (C) mixed patterns, which consists of more than two types of pulse notes or different types of notes such as pulse and frequency bands (Fig. 3). For calling songs of mixed patterns, a song sample was easily defined by examining the smallest stereotypic repetition of similar elements on the spectrograms (a repetition song). However, for most calling songs consisting of tonal band patterns and pulse trains patterns, the clear separation of individual calling songs from recordings of the automated digital systems was rare. Therefore, we cropped a 15–30 sec long calling sound of good quality as a sample from each recording for those species (a cropped song). In addition, we adjusted the low cut frequency for each species to remove the noise before making parameter measurements on the spectrograms. The sample unit and low cut frequency for each species were set as follows: a cropped song and 3 kHz low cut frequency for C*r. atrata* (S1 Sound), a cropped song and 3 kHz low cut frequency for *Ch. ochracea* (S2 Sound), a cropped song and 1 kHz low cut frequency for *S. watanabei* (S3 Sound), a repetition song and 6 kHz low cut frequency for *Pl. takasagona* (S4 Sound), a cropped song and 1.5 kHz low cut frequency for *Cr. holsti* (S5 Sound), a cropped song and 2 kHz low cut frequency for *Cr. takasagona* (S6 Sound), a cropped song and 2 kHz low cut frequency for *T. viridis* (S7 Sound), a repetition song and 3 kHz low cut frequency for *L. sakaii* (S8 Sound); a repetition song and 1 kHz low cut frequency for *E. gina* (S9 Sound), a repetition song and 5 kHz low cut frequency for *Mo. formosana* (S10 Sound), a repetition song and 1.5 kHz low cut frequency for *Me. opalifera* (S11 Sound), a repetition song and 1 kHz low cut frequency for *Po. linearis* (S12 Sound), and a repetition song and 1 kHz low cut frequency for *T. taipinensis* (S13 Sound).

We focused primarily on frequency features of calling songs for the interspecific acoustic comparison. The five frequency-related variables were measured based on the mean value for all spectra between the start and end of each song sample using the Automatic Parameter Measurements function in Avisoft-SASLab Pro. The variables were as follows: (1) peak frequency (PF: the frequency of the maximum amplitude of the spectrum; (2) quartile 25% (Q1: 25% of the total energy is below this frequency); (3) quartile 50% (Q2: the mean frequency of the spectrum, 50% of the total energy is below this frequency); (4) quartile 75% (Q3: 75% of the total energy is below this frequency); and (5) the pureness of the sound (the distance between Q3 and Q1) (Tables E and F in S1 File). Interspecific acoustic comparisons were then examined using a principal component analysis (PCA) on those five variables after a normalized transformation of each variable to have a mean of zero and unit standard deviation (software PRIMER 6, version 6.1.5). We retained all components with eigenvalues greater than one.

## Results

### Temporal partitioning of calling activity

Neither city assemblages nor mountain assemblages exhibited seasonal partitioning. No significant seasonal segregations (Spearman correlation coefficient r_s_ < 0, P < 0.05) were found between any two species at the mountain site or the two city sites (Tables Gand H in S1 File, Figures C and D in S2 File). However, there was evidence of diel partitioning among 8 species at the SP mountain site. Significant diel activity segregations were found in 9 of 55 species pairs at the SP mountain site (r_s_ < 0, P < 0.05) (Table G in S1 File, Figure A in S2 File). In addition, no significant relationship was found when regressing seasonal correlations with diel correlations at the SP mountain site (F_1,53_ = 2.34, P = 0.13, n = 55). At the two city sites, CC and SO, no significant diel calling segregations (r_s_ < 0, P < 0.05) were found among the three cicada species (Table H in S1 File, Figure B in S2 File).

### Acoustic feature partitioning

**Figure 4:**
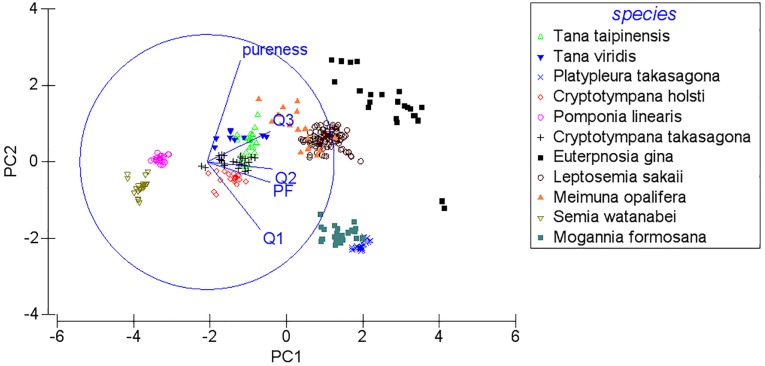
A bi-dimensional plotting of the principal component analysis using the five acoustic variables (PF, Q1, Q2, Q3, pureness) on spectrograms of cicada calling songs at the SP mountain site of Taiwan.

The SP mountain site had 11 cicada species in 2011. We retained two principal components with eigenvalues greater than one, and they accounted for 98.3% of the total variation (PC1: eigenvalue 3.75, 74.9% variation explained; PC2: eigenvalue 1.17, 23.4% variation explained). Four species pairs out of 55 pairs had acoustic features that overlapped on the plot of PC1 against PC2 (Fig. 4). They were *L. sakaii*-*Me. opalifera*; *T. taipinensis*-*T. viridis*; *Cr. takasagona*-*T. taipinensis*; and *Pl. takasagona*-*Mo. formosana*. Among the species pairs with similar acoustic frequency features, only the species pair *Cr. takasagona*-*T. taipinensis* showed a significant diel segregation (r_s_ < 0, P < 0.01) (Table G in S1 File). The congeneric and spectrally overlapping *T. taipinensis*-*T. viridis* species pair, even demonstrated significantly positive correlations of both seasonal and diel activity patterns (r_s_ > 0, P < 0.01) (Table G in S1 File). Further examination of the spectrograms of calling songs of this congeneric species pair found distinct time-frequency patterns. *T. taipinensis* had calling songs of mixed patterns, whereas *T. viridis* had calling songs of pulse trains patterns (Fig. 2). *Pl. takasagona* and *Mo. formosana* also had distinctly different time-frequency patterns of calling songs. The calling songs of *Pl. takasagona* were classified as tonal band patterns (Fig. 1), whereas those of *Mo. formosana* were classified as mixed patterns (Fig. 3). Although *L. sakaii* and *Me. opalifera* had similar spectral features and their calling songs were classified as mixed patterns, their calling songs consisted of different number of note types. The calling songs of *L. sakaii* consisted of two note types, whereas those of *Me. opalifera* consisted of more than four note types (Fig. 3).

To investigate the overall relationships between temporal partitioning and spectral partitioning at the SP site, we used the averages of PC1 and PC2 for each species to calculate the acoustic distance between two species. Neither seasonal correlations nor diel correlations had significant relationships with these acoustic distances (seasonal: F_1,53_ = 0.3, P = 0.58; diel: F_1,53_ = 0.02, P = 0.9).

**Figure 5:**
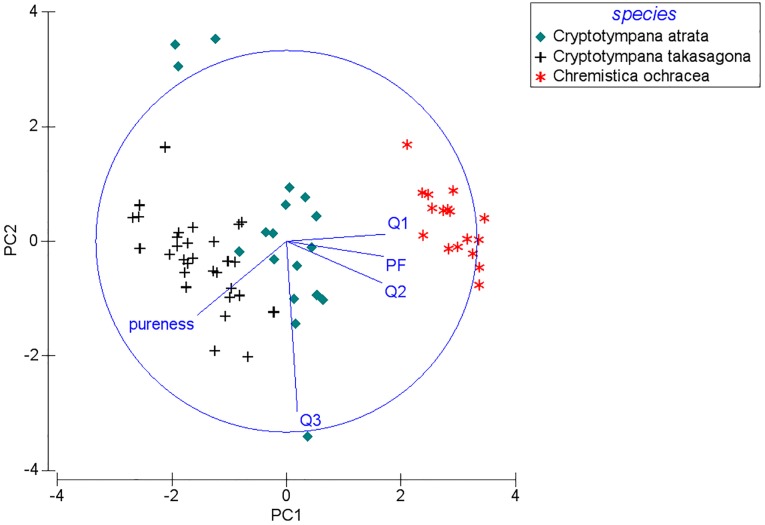
A bi-dimensional plotting of the principal component analysis using the five acoustic variables (PF, Q1, Q2, Q3, pureness) on spectrograms of cicada calling songs at the two city sites, CC and SO of Taiwan.

At the two city sites with three cicada species, two principal components were retained that accounted for 98.5% of the total variation (PC1: eigenvalue 3.69, 73.7% variation explained; PC2: eigenvalue 1.24, 24.8% variation explained). When plotting PC1 against PC2, the two congeneric species *Cr. atrata* and *Cr. takasagona* had overlapping spectral features (Fig. 5). This congeneric species pair synchronized their calling activity significantly at both the seasonal and diel scales at the CC site (P < 0.05), but not at the SO site (Table H in S1 File). However, *Cr. atrata* and *Cr. takasagona* had distinctly different time-frequency patterns of calling songs, with tonal band patterns for *Cr. atrata* (Fig. 1) and pulse trains patterns for *Cr. takasagona* (Fig. 2).

### Comparisons of calling activity patterns between city and mountain environments

For the common species *Cr. takasagona*, which was found in both city and mountain environments, the seasonal and diel calling activity patterns were both significantly similar across sites and across environments (seasonal calling activity patterns: SO-CC, r_s_ = 0.73, P < 0.01; SO-SP, r_s_ = 0.73, P < 0.01; CC-SP, r_s_ = 0.78, P < 0.01)(diel calling activity patterns: SO-CC: r_s_ = 0.92, P < 0.01; SO-SP: r_s_ = 0.87, P < 0.01; CC-SP: r_s_ = 0.92, P < 0.01) (Figures B and D in S2 File).

For *Cr. atrata* and *Ch. ochracea*, which were found at the two city sites, the seasonal calling activity patterns of each species were also significantly similar across sites (r_s_ > 0, P < 0.05) (Table H in S1 File). However, similar diel calling activity patterns across sites were only found in *Ch. ochracea* (r_s_ = 0.74, P < 0.01), not in *Cr. atrata* (r_s_ = -0.19, P = 0.34) (Figures B and D in S2 File).

## Discussion

The present study provides the first comparison of the spectral and temporal partitioning of cicada assemblages in city and mountain environments. We also compared the calling activity patterns of the common species between sites and between environments to investigate their consistency.

We found fewer cicada species and recorded higher anthropogenic noise at the city study sites than the mountain study site. These findings suggest that our city cicada assemblages have suffered more from selective pressures of anthropogenic noise and less from selective pressures of interspecific masking in comparison with mountain cicada assemblages. The effect of interspecific masking in our mountain site was similarly strong relative to that found in a Mexican rainforest, which included 9 cicada species [17].

Although city and mountain cicada assemblages were subject to significantly different levels of selective pressures of anthropogenic or interspecific noise, we found that they exhibited a convergent partitioning strategy. Spectral overlap was low in both environments. Most sympatric cicada species differed in the frequency features of their calling songs. Therefore, masking interference can be reduced by the partitioning of the frequency domain [4, 8, 32]. However, for sympatric species with an overlap in song frequency, avoidance of acoustic interference must incorporate features of the temporal domain; that is, cicada species with overlapping song frequency should have songs of different time-frequency patterns to reduce masking interference. We did observed distinct time-frequency patterns of calling songs for each species. This distinction was particularly important for separating calling songs of congeneric species. In our study, the congeneric species pairs, whether in city or mountain environments, all had overlaps in the frequency domain. We speculated that the overlaps in the frequency domain of congeneric cicadas might be due to similar sound production structures [14], resulting in constraints on song frequency range. Although it is easy to differentiate congeneric cicada species by hearing their songs and examining their distinct time-frequency patterns on spectrograms, we failed to provide quantitative evidence to distinguish congeneric cicada species in the present study. One possible explanation for our failure is that we did not measure features of the temporal domain in our acoustic analysis. We only measured features of the frequency domain for interspecific acoustic comparisons because it was difficult to extract common reliable features of the temporal domain from diverse time-frequency patterns, such as tonal bands or pulse trains. The other possible explanations for our failure in quantitatively distinguishing songs of congeneric cicadas might be the limitation of our spectrogram analysis, which is based on the Fourier transform, a linear system analysis. Since Benko and Perc [33–34] have demonstrated that sounds of insects, such as the Asian cicada (*Tosena depicta*) and katydid (*Neoconocephalus robustus*), possess clear markers of deterministic chaos, we suggested that a nonlinear analysis, such as the determination of the maximal Lyapunov exponent, might be beneficial to distinguish songs of congeneric cicadas quantitatively.

Regarding the temporal partitioning, we found no evidence of seasonal segregation between species at both environments. There were some cases (9/55) of diel segregation between species at the mountain sites and none at the city sites. In general, temporal partitioning was not notable for cicada assemblages, as has been found for cricket assemblages as well [35, 36]. Additionally, in contrast to partitioning, we found more cases of significant synchronization in calling activity between cicada species at both seasonal and diel scales. These results might suggest that temporal patterns of calling activity of ecologically similar species, but not congeners, are molded by shared responses to common external factors, such as noise or predation, which may result in more temporal overlap [19]. For congeneric species pairs in our study, such as *T. taipinensis*-*T. viridis* and *Cr. atrata*-*Cr. takasagona*, calling synchrony and spectral overlapping may indicate the possibility of a social communication network between closely related species, as suggested by Tobias et al. [37].

In the present study, calling activity patterns, whether at seasonal or diel scales, were also significantly similar between city and mountain environments for the common species, *Cr. takasagona*, and between sites for the city cicada species, *Ch. ochracea*. The other city cicada species, *Cr. atrata*, did not have similar diel calling patterns across sites (Table B in S1 File). The case of *Cr. atrata* might be because we only monitored very few sections of callings in two days for the whole year at the SO city site compared to those at the CC city site. The consistency of seasonal and diel calling patterns across sites and across environments may suggest that temporal patterns are more evolutionarily constrained by species endogenous rhythmicity [19]. Closely related species are thus constrained by phylogenetic endogenous rhythmicity and have similar temporal activity patterns.

In our study, the three city cicada species in the anthropogenic noise environment had tonal bands or pulse trains but not mixed patterns. We speculated that cicada species with songs of simple time-frequency patterns, such as pulse trains or tonal bands, may have been selected in city environments because they can shift their song frequency easily to adapt to higher levels of anthropogenic noise [30]. Alternatively, there might be other forces, such as habitats, selecting those city cicada species that happen to have simple time-frequency patterns. Further acoustic studies on city cicada assemblages are needed to test the two alternative explanations.

In conclusion, we found no marked temporal partitioning of cicada assemblages in both city and mountain environments. All cicada species had distinct time-frequency patterns of calling songs, and most species could be easily distinguished by acoustic frequency. Spectral overlap levels were low, suggesting that spectral partitioning could be the major strategy used to avoid acoustic interference in both city and mountain cicada assemblages. Additionally, the temporal calling patterns of species were consistent across sites and environments at both seasonal and diel scales.

## Supporting Information

S1 File This file contains Tables A-H. Table A, Diel calling activity of each cicada species at the SP mountain site. Table B, Diel calling activity of each cicada species at the two city sites, SO and CC. Table C, Seasonal calling activity of each cicada species at the SP mountain site. Table D, Seasonal calling activity of each cicada species at the two city sites, SO and CC. Table E, Descriptive statistics of acoustic measurements on spectrograms of calling songs of each cicada species at the SP mountain site. Table F, Descriptive statistics of acoustic measurements on spectrograms of calling songs of each cicada species at the two city sites, SO and CC. Table G, Spearman rank correlation coefficients (r_s_) of calling activities between species and their p-values in parentheses at the SP mountain site. Upper triangular part of the matrix: diel calling activity, n = 28. Lower triangular part of the matrix: seasonal calling activity, n = 24. Bolded numbers indicated significantly negative correlations at P < 0.05. Table H, Spearman rank correlation coefficients (r_s_) of calling activities between species and their p-values in parentheses at the two city sites, SO and CC. Upper triangular part of the matrix: diel calling activity, n = 28. Lower triangular part of the matrix: seasonal calling activity, n = 24.
(DOCX)

S2 File This file contains Figures A-D. Figure A, Diel calling activity pattern of each cicada species at the SP mountain site. Figure B, Diel calling activity pattern of each cicada species at the two city sites, SO and CC. Figure C, Semimonthly calling activity pattern of each cicada species at the SP mountain site. Figure D, Semimonthly calling activity pattern of each cicada species at the two city sites, SO and CC.
(DOCX)

S1 Sound A calling song example of *Cryptotympana atrata*. (WAV)

S2 Sound A calling song example of *Chremistica ochracea*. (WAV)

S3 Sound A calling song example of *Semia watanabei*. (WAV)

S4 Sound A calling song example of *Platypleura takasagona*. (WAV)

S5 Sound A calling song example of *Cryptotympana holsti*. (WAV)

S6 Sound A calling song example of *Cryptotympana takasagona*. (WAV)

S7 Sound A calling song example of *Tanna viridis*. (WAV)

S8 Sound A calling song example of *Leptosemia sakaii*. (WAV)

S9 Sound A calling song example of *Euterpnosia gina*. (WAV)

S10 Sound A calling song example of *Mogannia formosana*. (WAV)

S11 Sound A calling song example of *Meimuna opalifera*. (WAV)

S12 Sound A calling song example of *Pomponia linearis*. (WAV)

S13 Sound A calling song example of *Tanna taipinensis*. (WAV)
