# Supplementary material for: Acoustic and Temporal Partitioning of Cicada Assemblages in City and Mountain Environments
Source: PLoS One. 2015 Jan 15;10(1):e0116794. doi: 10.1371/journal.pone.0116794 (PMC4295890; doi:10.1371/journal.pone.0116794)
Supplement: S1 File — Table A, Diel calling activity of each cicada species at the SP mountain site. Table B, Diel calling activity of each cicada species at the two city sites, SO and CC. Table C, Seasonal calling activity of each cicada species at the SP mountain site. Table D, Seasonal calling activity of each cicada species at the two city sites, SO and CC. Table E, Descriptive statistics of acoustic measurements on spectrograms of calling songs of each cicada species at the SP mountain site. Table F, Descriptive statistics of acoustic measurements on spectrograms of calling songs of each cicada species at the two city sites, SO and CC. Table G, Spearman rank correlation coefficients (rs) of calling activities between species and their p-values in parentheses at the SP mountain site. Upper triangular part of the matrix: diel calling activity, n = 28. Lower triangular part of the matrix: seasonal calling activity, n = 24. Bolded numbers indicated significantly negative correlations at P < 0.05. Table H, Spearman rank correlation coefficients (rs) of calling activities between species and their p-values in parentheses at the two city sites, SO and CC. Upper triangular part of the matrix: diel calling activity, n = 28. Lower triangular part of the matrix: seasonal calling activity, n = 24. (DOCX) [file pone.0116794.s001.docx]

Table A. Diel calling activity of each cicada species at the SP mountain site.

| Time\Species | *Cr. h.* | *Cr. t.* | *E. g.* | *L. s.* | *Mo. f.* | *Me. o.* | *Po. l.* | *Pl. t.* | *S. w.* | *T. t.* | *T. v.* |
| --- | --- | --- | --- | --- | --- | --- | --- | --- | --- | --- | --- |
| 05:30 | 0.0023 | 0.0000 | 0.0000 | 0.0057 | 0.0000 | 0.0000 | 0.0354 | 0.7059 | 0.0000 | 0.1270 | 0.0769 |
| 06:00 | 0.0151 | 0.0000 | 0.0024 | 0.0000 | 0.0000 | 0.0000 | 0.0000 | 0.5176 | 0.0000 | 0.0000 | 0.0000 |
| 06:30 | 0.1012 | 0.0000 | 0.0000 | 0.0314 | 0.0000 | 0.0200 | 0.0205 | 0.5765 | 0.0000 | 0.0000 | 0.0000 |
| 07:00 | 0.2779 | 0.0185 | 0.0145 | 0.0829 | 0.0000 | 0.0215 | 0.0087 | 0.6000 | 0.0208 | 0.0000 | 0.0000 |
| 07:30 | 0.5070 | 0.0185 | 0.0193 | 0.0971 | 0.0254 | 0.0923 | 0.0339 | 0.4882 | 0.0000 | 0.0000 | 0.0000 |
| 08:00 | 0.5593 | 0.0796 | 0.0458 | 0.0514 | 0.0333 | 0.2585 | 0.0465 | 0.5824 | 0.0000 | 0.0000 | 0.0077 |
| 08:30 | 0.6372 | 0.2870 | 0.1000 | 0.1086 | 0.0810 | 0.4400 | 0.0488 | 0.5059 | 0.0063 | 0.0000 | 0.0000 |
| 09:00 | 0.6558 | 0.2870 | 0.1422 | 0.1143 | 0.0857 | 0.5354 | 0.0386 | 0.6235 | 0.0167 | 0.0000 | 0.0000 |
| 09:30 | 0.6384 | 0.2574 | 0.1663 | 0.1086 | 0.1095 | 0.5092 | 0.0661 | 0.4412 | 0.0000 | 0.0000 | 0.0000 |
| 10:00 | 0.6570 | 0.1685 | 0.2386 | 0.1400 | 0.1651 | 0.5185 | 0.0504 | 0.4824 | 0.0208 | 0.0000 | 0.0000 |
| 10:30 | 0.6070 | 0.2056 | 0.2205 | 0.0829 | 0.1635 | 0.5477 | 0.0543 | 0.6176 | 0.0333 | 0.0000 | 0.0000 |
| 11:00 | 0.5814 | 0.1389 | 0.2651 | 0.1200 | 0.1857 | 0.5477 | 0.0787 | 0.5471 | 0.0213 | 0.0000 | 0.0000 |
| 11:30 | 0.4919 | 0.2037 | 0.2651 | 0.1824 | 0.1000 | 0.5462 | 0.1055 | 0.5118 | 0.0396 | 0.0000 | 0.0000 |
| 12:00 | 0.4128 | 0.1815 | 0.3108 | 0.2457 | 0.0714 | 0.5354 | 0.1150 | 0.5000 | 0.0479 | 0.0000 | 0.0000 |
| 12:30 | 0.3837 | 0.1519 | 0.2301 | 0.4114 | 0.0889 | 0.5462 | 0.1567 | 0.4765 | 0.0417 | 0.0009 | 0.0000 |
| 13:00 | 0.3733 | 0.0407 | 0.2048 | 0.4800 | 0.0571 | 0.5585 | 0.2024 | 0.3294 | 0.0833 | 0.0000 | 0.0000 |
| 13:30 | 0.3105 | 0.0370 | 0.2241 | 0.4886 | 0.0206 | 0.5708 | 0.2354 | 0.4294 | 0.0125 | 0.0432 | 0.0000 |
| 14:00 | 0.2860 | 0.0000 | 0.2181 | 0.3657 | 0.0381 | 0.5692 | 0.2913 | 0.3353 | 0.0292 | 0.0081 | 0.0000 |
| 14:30 | 0.2837 | 0.0167 | 0.2207 | 0.4314 | 0.0159 | 0.4954 | 0.2677 | 0.4706 | 0.0875 | 0.0423 | 0.0000 |
| 15:00 | 0.2047 | 0.0111 | 0.1928 | 0.4571 | 0.0016 | 0.4692 | 0.3906 | 0.4529 | 0.0896 | 0.0432 | 0.0500 |
| 15:30 | 0.1279 | 0.0370 | 0.1627 | 0.5314 | 0.0000 | 0.3985 | 0.4205 | 0.4941 | 0.0708 | 0.0802 | 0.0000 |
| 16:00 | 0.1023 | 0.0185 | 0.1446 | 0.4486 | 0.0000 | 0.2723 | 0.5531 | 0.5412 | 0.1000 | 0.0793 | 0.0192 |
| 16:30 | 0.0535 | 0.0000 | 0.1349 | 0.4714 | 0.0000 | 0.2600 | 0.5984 | 0.4471 | 0.0167 | 0.0495 | 0.0615 |
| 17:00 | 0.0430 | 0.0056 | 0.0964 | 0.4400 | 0.0000 | 0.2200 | 0.6164 | 0.4412 | 0.0313 | 0.0865 | 0.0269 |
| 17:30 | 0.0116 | 0.0000 | 0.0325 | 0.3714 | 0.0000 | 0.2031 | 0.7289 | 0.2235 | 0.0229 | 0.1018 | 0.0538 |
| 18:00 | 0.0012 | 0.0000 | 0.0217 | 0.2114 | 0.0000 | 0.0477 | 0.7500 | 0.1176 | 0.0104 | 0.1225 | 0.1192 |
| 18:30 | 0.0000 | 0.0000 | 0.1542 | 0.0114 | 0.0000 | 0.0000 | 0.6711 | 0.0000 | 0.2854 | 0.1414 | 0.1500 |
| 19:00 | 0.0000 | 0.0000 | 0.0000 | 0.0000 | 0.0000 | 0.0000 | 0.0000 | 0.0000 | 0.0000 | 0.0000 | 0.0000 |

Table B. Diel calling activity of each cicada species at the two city sites, SO and CC.

| Time\Species_Site | *Cr. a.*_SO | *Ch. o.*_SO | *Cr. t.*_SO | *Cr. t.*_CC | *Cr. a*._CC | *Ch. o.*_CC |
| --- | --- | --- | --- | --- | --- | --- |
| 05:30 | 0.0000 | 0.0000 | 0.0127 | 0.0000 | 0.0000 | 0.0062 |
| 06:00 | 0.0000 | 0.0135 | 0.0509 | 0.0064 | 0.0191 | 0.0000 |
| 06:30 | 0.0000 | 0.0558 | 0.1945 | 0.0904 | 0.1106 | 0.0400 |
| 07:00 | 0.0000 | 0.0692 | 0.4255 | 0.2894 | 0.2404 | 0.0523 |
| 07:30 | 0.0000 | 0.0712 | 0.5327 | 0.4117 | 0.2915 | 0.0969 |
| 08:00 | 0.0000 | 0.1135 | 0.6000 | 0.5936 | 0.3809 | 0.0800 |
| 08:30 | 0.0000 | 0.1250 | 0.7164 | 0.6872 | 0.3128 | 0.1108 |
| 09:00 | 0.0000 | 0.0846 | 0.7685 | 0.6681 | 0.4383 | 0.1338 |
| 09:30 | 0.0000 | 0.1558 | 0.7745 | 0.6947 | 0.3830 | 0.0862 |
| 10:00 | 0.0000 | 0.0755 | 0.8073 | 0.6074 | 0.3298 | 0.0908 |
| 10:30 | 0.0000 | 0.1472 | 0.8073 | 0.6232 | 0.2766 | 0.0769 |
| 11:00 | 0.0000 | 0.0566 | 0.8400 | 0.6842 | 0.2511 | 0.0569 |
| 11:30 | 0.0000 | 0.0415 | 0.8618 | 0.6179 | 0.1702 | 0.0446 |
| 12:00 | 0.0000 | 0.0925 | 0.8291 | 0.5453 | 0.2787 | 0.0815 |
| 12:30 | 0.0000 | 0.0698 | 0.7873 | 0.5505 | 0.3255 | 0.1415 |
| 13:00 | 0.0000 | 0.1019 | 0.7455 | 0.4779 | 0.3255 | 0.0923 |
| 13:30 | 0.0000 | 0.1057 | 0.6182 | 0.4074 | 0.2617 | 0.0862 |
| 14:00 | 0.0000 | 0.1019 | 0.4000 | 0.3842 | 0.2191 | 0.0538 |
| 14:30 | 0.3500 | 0.0811 | 0.3618 | 0.2611 | 0.2404 | 0.0554 |
| 15:00 | 0.0000 | 0.0577 | 0.2818 | 0.1568 | 0.1851 | 0.0677 |
| 15:30 | 0.1500 | 0.0846 | 0.1818 | 0.1000 | 0.1064 | 0.0615 |
| 16:00 | 0.0000 | 0.0096 | 0.0600 | 0.0884 | 0.1213 | 0.0385 |
| 16:30 | 0.0000 | 0.0615 | 0.0364 | 0.0516 | 0.0745 | 0.0708 |
| 17:00 | 0.1000 | 0.0885 | 0.0600 | 0.0821 | 0.0660 | 0.1323 |
| 17:30 | 0.0000 | 0.1077 | 0.0091 | 0.0358 | 0.0511 | 0.1615 |
| 18:00 | 0.0000 | 0.2077 | 0.0000 | 0.0526 | 0.1043 | 0.2985 |
| 18:30 | 0.0000 | 0.3577 | 0.0000 | 0.0789 | 0.0766 | 0.7385 |
| 19:00 | 0.0000 | 0.0000 | 0.0000 | 0.0000 | 0.0000 | 0.0000 |

Table C. Seasonal calling activity of each cicada species at the SP mountain site.

| Season\Species | *Cr. h.* | *Cr. t.* | *E. g.* | *L. s.* | *Mo. f.* | *Me. o.* | *Po. l.* | *Pl. t.* | *S. w.* | *T. t.* | *T. v.* |
| --- | --- | --- | --- | --- | --- | --- | --- | --- | --- | --- | --- |
| 01/01/2011 | 0.00000 | 0.00000 | 0.00000 | 0.00000 | 0.00000 | 0.00000 | 0.00000 | 0.00000 | 0.00000 | 0.00000 | 0.00000 |
| 01/16/2011 | 0.00000 | 0.00000 | 0.00000 | 0.00000 | 0.00000 | 0.00000 | 0.00000 | 0.00000 | 0.00000 | 0.00000 | 0.00000 |
| 02/01/2011 | 0.00000 | 0.00000 | 0.00000 | 0.00000 | 0.00000 | 0.00000 | 0.00000 | 0.00000 | 0.00000 | 0.00000 | 0.00000 |
| 02/16/2011 | 0.00000 | 0.00000 | 0.00000 | 0.00000 | 0.00000 | 0.00000 | 0.00000 | 0.00000 | 0.00000 | 0.00000 | 0.00000 |
| 03/01/2011 | 0.00000 | 0.00000 | 0.00000 | 0.00000 | 0.00000 | 0.00000 | 0.00000 | 0.00000 | 0.00000 | 0.00000 | 0.00000 |
| 03/16/2011 | 0.00000 | 0.00000 | 0.00000 | 0.00000 | 0.00000 | 0.00000 | 0.00000 | 0.00000 | 0.00000 | 0.00000 | 0.00000 |
| 04/01/2011 | 0.00000 | 0.00000 | 0.09929 | 0.00000 | 0.00000 | 0.00000 | 0.00000 | 0.00000 | 0.00000 | 0.00000 | 0.00000 |
| 04/16/2011 | 0.00000 | 0.00000 | 0.20143 | 0.00000 | 0.00000 | 0.00000 | 0.00000 | 0.00000 | 0.01534 | 0.00000 | 0.00000 |
| 05/01/2011 | 0.00000 | 0.00000 | 0.21571 | 0.07833 | 0.03012 | 0.00000 | 0.00000 | 0.00000 | 0.03619 | 0.01202 | 0.00000 |
| 05/16/2011 | 0.00089 | 0.00000 | 0.09978 | 0.33856 | 0.05000 | 0.00000 | 0.19821 | 0.07210 | 0.04922 | 0.03259 | 0.01931 |
| 06/01/2011 | 0.30750 | 0.00000 | 0.14210 | 0.13652 | 0.06655 | 0.00000 | 0.24143 | 0.42738 | 0.02429 | 0.02655 | 0.02145 |
| 06/16/2011 | 0.50976 | 0.00000 | 0.05262 | 0.01074 | 0.03952 | 0.00000 | 0.22429 | 0.00000 | 0.02960 | 0.01167 | 0.01443 |
| 07/01/2011 | 0.50119 | 0.04083 | 0.01381 | 0.00000 | 0.02637 | 0.00000 | 0.28619 | 0.00000 | 0.00373 | 0.03476 | 0.02662 |
| 07/16/2011 | 0.31429 | 0.06797 | 0.00000 | 0.00000 | 0.01657 | 0.00000 | 0.28839 | 0.00000 | 0.00000 | 0.03705 | 0.03132 |
| 08/01/2011 | 0.08987 | 0.08190 | 0.00000 | 0.00000 | 0.03127 | 0.00000 | 0.37589 | 0.00000 | 0.00000 | 0.02363 | 0.01433 |
| 08/16/2011 | 0.03841 | 0.03627 | 0.00000 | 0.00000 | 0.02949 | 0.01406 | 0.43633 | 0.00000 | 0.00000 | 0.02785 | 0.00971 |
| 09/01/2011 | 0.01083 | 0.04855 | 0.00000 | 0.00000 | 0.00156 | 0.38619 | 0.17857 | 0.00000 | 0.00000 | 0.05667 | 0.01667 |
| 09/16/2011 | 0.00627 | 0.09270 | 0.00000 | 0.00000 | 0.00000 | 0.55881 | 0.02914 | 0.00000 | 0.00000 | 0.03262 | 0.00000 |
| 10/01/2011 | 0.00170 | 0.12304 | 0.00000 | 0.00000 | 0.00000 | 0.34476 | 0.00908 | 0.00000 | 0.00000 | 0.01798 | 0.00000 |
| 10/16/2011 | 0.00000 | 0.01975 | 0.00000 | 0.00000 | 0.00000 | 0.11585 | 0.00000 | 0.00000 | 0.00000 | 0.00000 | 0.00000 |
| 11/01/2011 | 0.00000 | 0.02833 | 0.00000 | 0.00000 | 0.00000 | 0.00238 | 0.00000 | 0.00000 | 0.00000 | 0.00000 | 0.00000 |
| 11/16/2011 | 0.00000 | 0.00000 | 0.00000 | 0.00000 | 0.00000 | 0.00000 | 0.00000 | 0.00000 | 0.00000 | 0.00000 | 0.00000 |
| 12/01/2011 | 0.00000 | 0.00000 | 0.00000 | 0.00000 | 0.00000 | 0.00000 | 0.00000 | 0.00000 | 0.00000 | 0.00000 | 0.00000 |
| 12/16/2011 | 0.00000 | 0.00000 | 0.00000 | 0.00000 | 0.00000 | 0.00000 | 0.00000 | 0.00000 | 0.00000 | 0.00000 | 0.00000 |

Table D. Seasonal calling activity of each cicada species at the two city sites, SO and CC.

| Season\Species_Site | *Cr. a.*_SO | *Ch. o.*_SO | *Cr. t.*_SO | *Cr. t.*_CC | *Cr. a.*_CC | *Ch. o.*_CC |
| --- | --- | --- | --- | --- | --- | --- |
| 01/01/2011 | 0.00000 | 0.00000 | 0.00000 | 0.00000 | 0.00000 | 0.00000 |
| 01/16/2011 | 0.00000 | 0.00000 | 0.00000 | 0.00000 | 0.00000 | 0.00000 |
| 02/01/2011 | 0.00000 | 0.00000 | 0.00000 | 0.00000 | 0.00000 | 0.00000 |
| 02/16/2011 | 0.00000 | 0.00000 | 0.00000 | 0.00000 | 0.00000 | 0.00000 |
| 03/01/2011 | 0.00000 | 0.00000 | 0.00000 | 0.00000 | 0.00000 | 0.00000 |
| 03/16/2011 | 0.00000 | 0.00000 | 0.00000 | 0.00000 | 0.00000 | 0.00000 |
| 04/01/2011 | 0.00000 | 0.00000 | 0.00000 | 0.00000 | 0.00000 | 0.00000 |
| 04/16/2011 | 0.00000 | 0.00000 | 0.00000 | 0.00000 | 0.00000 | 0.00000 |
| 05/01/2011 | 0.00000 | 0.00000 | 0.00000 | 0.00000 | 0.00000 | 0.00000 |
| 05/16/2011 | 0.00000 | 0.00000 | 0.00000 | 0.00000 | 0.00000 | 0.00000 |
| 06/01/2011 | 0.00000 | 0.00000 | 0.00000 | 0.07000 | 0.02381 | 0.09048 |
| 06/16/2011 | 0.00286 | 0.46510 | 0.00000 | 0.38488 | 0.25310 | 0.20048 |
| 07/01/2011 | 0.00000 | 0.16942 | 0.00000 | 0.34524 | 0.25238 | 0.06738 |
| 07/16/2011 | 0.00000 | 0.06853 | 0.00864 | 0.30937 | 0.12321 | 0.06741 |
| 08/01/2011 | 0.00000 | 0.05976 | 0.01253 | 0.16119 | 0.00381 | 0.02870 |
| 08/16/2011 | 0.00000 | 0.02076 | 0.01092 | 0.06540 | 0.00000 | 0.01250 |
| 09/01/2011 | 0.00000 | 0.00000 | 0.00932 | 0.20818 | 0.00000 | 0.00189 |
| 09/16/2011 | 0.00000 | 0.00000 | 0.00902 | 0.42470 | 0.00000 | 0.00000 |
| 10/01/2011 | 0.00000 | 0.00000 | 0.29863 | 0.64122 | 0.00000 | 0.00000 |
| 10/16/2011 | 0.00000 | 0.00000 | 0.62656 | 0.65776 | 0.00000 | 0.00000 |
| 11/01/2011 | 0.00000 | 0.00000 | 0.41762 | 0.35917 | 0.00000 | 0.00000 |
| 11/16/2011 | 0.00000 | 0.00000 | 0.20095 | 0.03202 | 0.00000 | 0.00000 |
| 12/01/2011 | 0.00000 | 0.00000 | 0.00000 | 0.00000 | 0.00000 | 0.00000 |
| 12/16/2011 | 0.00000 | 0.00000 | 0.00000 | 0.00000 | 0.00000 | 0.00000 |

Table E. Descriptive statistics of acoustic measurements on spectrograms of calling songs of each cicada species at the SP mountain site.

| Species | Statistics\Variable | PF | Q1 | Q2 | Q3 | pureness |
| --- | --- | --- | --- | --- | --- | --- |
| *Tana taipinensis* | n | 28 | 28 | 28 | 28 | 28 |
|  | Mean | 5371 | 4272 | 6075 | 8901 | 4629 |
|  | SD | 277 | 260 | 231 | 395 | 526 |
|  | C.V. | 0.052 | 0.061 | 0.038 | 0.044 | 0.114 |
| *Tana viridis* | n | 9 | 9 | 9 | 9 | 9 |
|  | Mean | 4646 | 3957 | 5831 | 8686 | 4729 |
|  | SD | 867 | 94 | 842 | 404 | 351 |
|  | C.V. | 0.187 | 0.024 | 0.144 | 0.047 | 0.074 |
| *Platypleura takasagona* | n | 30 | 30 | 30 | 30 | 30 |
|  | Mean | 9902 | 9291 | 10076 | 11090 | 1800 |
|  | SD | 111 | 85 | 110 | 280 | 247 |
|  | C.V. | 0.011 | 0.009 | 0.011 | 0.025 | 0.137 |
| *Cryptotympana holsti* | n | 20 | 20 | 20 | 20 | 20 |
|  | Mean | 5578 | 4553 | 5812 | 7332 | 2779 |
|  | SD | 356 | 252 | 314 | 395 | 318 |
|  | C.V. | 0.064 | 0.055 | 0.054 | 0.054 | 0.114 |
| *Pomponia linearis* | n | 24 | 24 | 24 | 24 | 24 |
|  | Mean | 3051 | 2583 | 3702 | 5200 | 2617 |
|  | SD | 184 | 105 | 171 | 200 | 162 |
|  | C.V. | 0.06 | 0.041 | 0.046 | 0.038 | 0.062 |
| *Cryptotympana takasagona* | n | 24 | 24 | 24 | 24 | 24 |
|  | Mean | 5153 | 4306 | 5944 | 7856 | 3550 |
|  | SD | 555 | 316 | 396 | 511 | 293 |
|  | C.V. | 0.108 | 0.073 | 0.067 | 0.065 | 0.083 |
| *Euterpnosia gina* | n | 23 | 23 | 23 | 23 | 23 |
|  | Mean | 10743 | 5514 | 10440 | 13683 | 8170 |
|  | SD | 1707 | 1426 | 1366 | 434 | 1261 |
|  | C.V. | 0.159 | 0.259 | 0.131 | 0.032 | 0.154 |
| *Leptosemia sakaii* | n | 107 | 107 | 107 | 107 | 107 |
|  | Mean | 7581 | 5784 | 8792 | 11695 | 5911 |
|  | SD | 691 | 219 | 421 | 431 | 396 |
|  | C.V. | 0.091 | 0.038 | 0.048 | 0.037 | 0.067 |
| *Meimuna opalifera* | n | 16 | 16 | 16 | 16 | 16 |
|  | Mean | 6274 | 5096 | 7871 | 11028 | 5932 |
|  | SD | 1063 | 704 | 620 | 472 | 669 |
|  | C.V. | 0.169 | 0.138 | 0.079 | 0.043 | 0.113 |
| *Semia watanabei* | n | 23 | 23 | 23 | 23 | 23 |
|  | Mean | 2953 | 2826 | 3105 | 3932 | 1106 |
|  | SD | 205 | 237 | 87 | 338 | 379 |
|  | C.V. | 0.069 | 0.084 | 0.028 | 0.086 | 0.343 |
| *Mogannia formosana* | n | 23 | 23 | 23 | 23 | 23 |
|  | Mean | 9177 | 8480 | 9401 | 10458 | 1978 |
|  | SD | 303 | 416 | 359 | 540 | 356 |
|  | C.V. | 0.033 | 0.049 | 0.038 | 0.052 | 0.18 |

Table F. Descriptive statistics of acoustic measurements on spectrograms of calling songs of each cicada species at the two city sites, SO and CC.

| Species | Statistics\Variable | PF | Q1 | Q2 | Q3 | pureness |
| --- | --- | --- | --- | --- | --- | --- |
| *Cryptotympana atrata* | n | 18 | 18 | 18 | 18 | 18 |
|  | Mean | 5637 | 5024 | 6324 | 7946 | 2921 |
|  | SD | 466 | 258 | 354 | 495 | 324 |
|  | C.V. | 0.083 | 0.051 | 0.056 | 0.062 | 0.111 |
| *Cryptotympana takasagona* | n | 30 | 30 | 30 | 30 | 30 |
|  | Mean | 5325 | 4356 | 6101 | 8042 | 3686 |
|  | SD | 412 | 209 | 181 | 215 | 184 |
|  | C.V. | 0.077 | 0.048 | 0.03 | 0.027 | 0.05 |
| *Chremistica ochracea* | n | 17 | 17 | 17 | 17 | 17 |
|  | Mean | 7080 | 6085 | 7096 | 7949 | 1864 |
|  | SD | 207 | 163 | 175 | 169 | 125 |
|  | C.V. | 0.029 | 0.027 | 0.025 | 0.021 | 0.067 |

Table G. Spearman rank correlation coefficients (r_s_) of calling activities between species and their p-values in parentheses at the SP mountain site. Upper triangular part of the matrix: diel calling activity, n = 28. Lower triangular part of the matrix: seasonal calling activity, n = 24. Bolded numbers indicated significantly negative correlations at P < 0.05.

| *Species* | *Cr. h.* | *Cr. t.* | *E. g.* | *L. s.* | *M. f.* | *M. o.* | *Po. l.* | *Pl. t.* | *S. w.* | *T. t.* | *T. v.* |
| --- | --- | --- | --- | --- | --- | --- | --- | --- | --- | --- | --- |
| *Cr. h.* | 1.00 | 0.90 | 0.55 | 0.04 | 0.91 | 0.68 | -0.32 | 0.40 | -0.05 | **-0.67** | **-0.61** |
|  |  | (<0.01) | (<0.01) | (0.84) | (<0.01) | (<0.01) | (0.10) | (0.04) | (0.80) | **(<0.01)** | **(<0.01)** |
| *Cr. t.* | 0.57 | 1.00 | 0.58 | 0.09 | 0.82 | 0.64 | -0.23 | 0.39 | 0.10 | **-0.58** | **-0.55** |
|  | (<0.01) |  | (<.01) | (0.63) | (<.01) | (<.01) | (0.24) | (0.04) | (0.61) | **(<0.01)** | **(<0.01)** |
| *E. g.* | 0.18 | -0.35 | 1.00 | 0.50 | 0.72 | 0.85 | 0.33 | -0.08 | 0.60 | -0.13 | -0.34 |
|  | (0.40) | (0.10) |  | (0.01) | (<0.01) | (<0.01) | (0.09 | (0.70 | (<0.01) | (0.50) | (0.08) |
| *L. s.* | 0.30 | -0.33 | 0.72 | 1.00 | 0.02 | 0.54 | 0.71 | -0.35 | 0.58 | 0.38 | 0.06 |
|  | (0.15) | (0.11) | (<0.01) |  | (0.92) | (<0.01) | (<0.01) | 0.07 | (<0.01) | (0.04) | (0.75) |
| *M. f.* | 0.76 | 0.15 | 0.52 | 0.73 | 1.00 | 0.77 | -0.21 | 0.22 | 0.06 | **-0.60** | **-0.56** |
|  | (<0.01) | (0.49) | (0.01) | (<0.01) |  | (<0.01) | (0.28) | (0.27) | (0.77) | **(<0.01)** | **(<0.01)** |
| *M. o.* | 0.19 | 0.71 | -0.36 | -0.25 | -0.13 | 1.00 | 0.19 | 0.002 | 0.38 | -0.27 | **-0.49** |
|  | (0.38) | (<0.01) | (0.09) | (0.23) | (0.54) |  | (0.33) | (0.99 | (0.05) | (0.16) | **(0.01)** |
| *Po. l.* | 0.96 | 0.60 | 0.13 | 0.27 | 0.78 | 0.20 | 1.00 | **-0.58** | 0.64 | 0.75 | 0.57 |
|  | (<0.01) | (<0.01) | (0.55) | (0.21) | (<0.01) | (0.34) |  | **(<0.01)** | (<0.01) | (<0.01) | (<0.01) |
| *Pl. t.* | 0.27 | -0.23 | 0.49 | 0.74 | 0.55 | -0.17 | 0.32 | 1.00 | -0.20 | **-0.40** | -0.25 |
|  | (0.20) | (0.29) | (0.02) | (<0.01) | (0.01) | (0.42) | (0.13) |  | (0.31) | **(0.04)** | (0.20) |
| *S. w.* | 0.33 | -0.29 | 0.90 | 0.85 | 0.67 | -0.32 | 0.27 | 0.57 | 1.00 | 0.35 | 0.12 |
|  | (0.12) | (0.17) | (<0.01) | (<0.01) | (<0.01) | (0.12) | (0.21) | (<0.01) |  | (0.07) | (0.54) |
| *T. t.* | 0.86 | 0.65 | 0.19 | 0.31 | 0.72 | 0.34 | 0.88 | 0.31 | 0.32 | 1.00 | 0.77 |
|  | (<0.01) | (<0.01) | (0.37) | (0.14) | (<0.01) | (0.10) | (<0.01) | (0.15) | (0.13) |  | (<0.01) |
| *T. v.* | 0.87 | 0.34 | 0.29 | 0.41 | 0.85 | -0.06 | 0.88 | 0.47 | 0.43 | 0.81 | 1.00 |
|  | (<0.01) | (0.10) | (0.17) | (0.05) | (<0.01) | (0.78) | (<0.01) | (0.02) | (0.03) | (<0.01) |  |

Table H. Spearman rank correlation coefficients (r_s_) of calling activities between species and their p-values in parentheses at the two city sites, SO and CC. Upper triangular part of the matrix: diel calling activity, n = 28. Lower triangular part of the matrix: seasonal calling activity, n = 24.

| *Species*_Site | *Cr. a.* _SO | *Ch. o.* _SO | *Cr. t.* _SO | *Cr. t.* _CC | *Cr. a.* _CC | *Ch. o.* _CC |
| --- | --- | --- | --- | --- | --- | --- |
| *Cr. a.* _SO | 1.00 | 0.04 | -0.17 | -0.16 | -0.19 | -0.02 |
|  |  | (0.84) | (0.40) | (0.42) | (0.34) | (0.92) |
| *Ch. o.* _SO | 0.49 | 1.00 | 0.10 | 0.33 | 0.38 | 0.74 |
|  | (0.02) |  | (0.62) | (0.08) | (0.04) | (<0.01) |
| *Cr. t.* _SO | -0.16 | 0.10 | 1.00 | 0.92 | 0.8 | 0.08 |
|  | (0.47) | (0.66) |  | (<0.01) | (<0.01) | (0.67) |
| *Cr. t.* _CC | 0.27 | 0.42 | 0.73 | 1.00 | 0.89 | 0.26 |
|  | (0.20) | (0.04) | (<0.01) |  | (<0.01) | (0.19) |
| *Cr. a.* _CC | 0.49 | 0.81 | -0.08 | 0.43 | 1.00 | 0.37 |
|  | (0.02) | (<0.01) | (0.72) | (0.03) |  | (0.054) |
| *Ch. o.* _CC | 0.43 | 0.81 | 0.07 | 0.47 | 0.88 | 1.00 |
|  | (0.04) | (<0.01) | (0.74) | (0.02) | (<0.01) |  |
