# Supplementary material for: Acoustic and Temporal Partitioning of Cicada Assemblages in City and Mountain Environments
Source: PLoS One. 2015 Jan 15;10(1):e0116794. doi: 10.1371/journal.pone.0116794 (PMC4295890; doi:10.1371/journal.pone.0116794)
Supplement: S2 File — Figure A, Diel calling activity pattern of each cicada species at the SP mountain site. Figure B, Diel calling activity pattern of each cicada species at the two city sites, SO and CC. Figure C, Semimonthly calling activity pattern of each cicada species at the SP mountain site. Figure D, Semimonthly calling activity pattern of each cicada species at the two city sites, SO and CC. (DOCX) [file pone.0116794.s002.docx]

Figure A. Diel calling activity pattern of each cicada species at the SP mountain site.

Figure B. Diel calling activity pattern of each cicada species at the two city sites, SO and CC.

Figure C. Semimonthly calling activity pattern of each cicada species at the SP mountain site.

Figure D. Semimonthly calling activity pattern of each cicada species at the two city sites, SO and CC.
